# Supplementary material for: Predicting visual function by interpreting a neuronal wiring diagram
Source: Nature. 2024 Oct 2;634(8032):113–23. doi: 10.1038/s41586-024-07953-5 (PMC11446822; doi:10.1038/s41586-024-07953-5)
Supplement: Supplementary file 6 — CRF and ERF predictions for individual TmY4 and TmY9 cells. Analogous to Supplementary Data 3, but for TmY target types. Shown are the top four monosynaptic pathways, the strongest pathway passing through each of the top ten intermediary types (ranking from Extended Data Fig. 7), and the trisynaptic pathway Tm1–TmY–Dm3–TmY (see the section entitled Prediction of spatial normalization). [file 41586_2024_7953_MOESM6_ESM.zip › DataS4/TmY9q/720575940637236287.pdf]

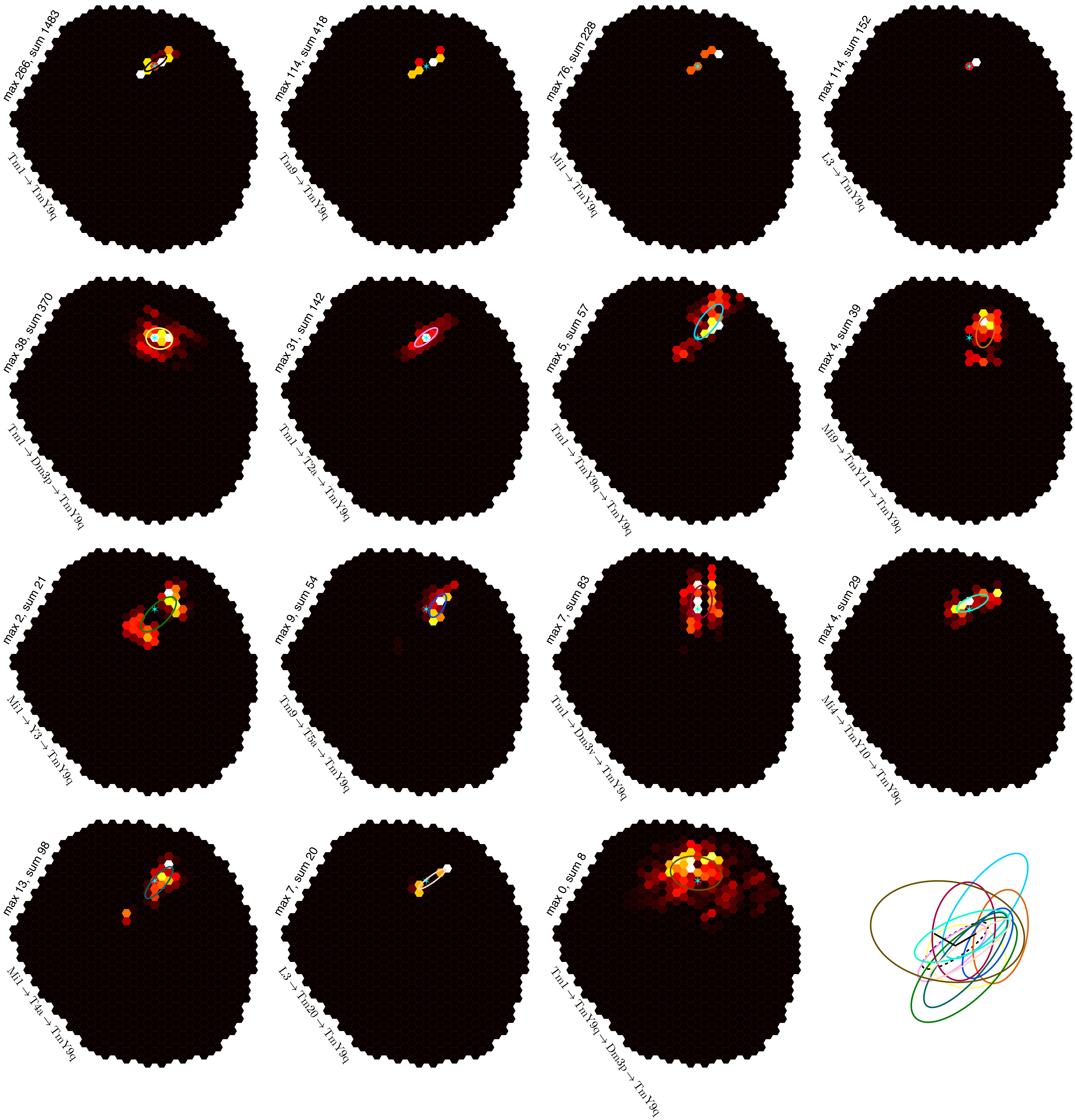
max 266, sum 1483
Tm1 → TmY9q
max 114, sum 418
Tm9 → TmY9q
max 76, sum 228
Mi1 → TmY9q
max 114, sum 152
L3 → TmY9q
max 38, sum 370
Tm1 → Dm3p → TmY9q
max 31, sum 142
Tm1 → T2a → TmY9q
max 5, sum 57
Tm1 → TmY9q → TmY9q
max 4, sum 39
Mi9 → TmY11 → TmY9q
max 2, sum 21
Mi1 → Y3 → TmY9q
max 9, sum 54
Tm9 → T5a → TmY9q
max 7, sum 83
Tm1 → Dm3v → TmY9q
max 4, sum 29
Mi4 → TmY10 → TmY9q
max 13, sum 98
Mi1 → T4a → TmY9q
max 7, sum 20
L3 → Tm20 → TmY9q
max 0, sum 8
Tm1 → TmY9q → Dm3p → TmY9q
